# 538 Lessons Learned: Helping Non-Burn Facilities Prepare for Burn Mass Casualty Disasters

**DOI:** 10.1093/jbcr/irae036.172

**Published:** 2024-04-17

**Authors:** Amy Steinert, Julia K Mendelson

**Affiliations:** Legacy Emanuel, Portland, OR; Oregon Burn Center, Portland, OR; Legacy Emanuel, Portland, OR; Oregon Burn Center, Portland, OR

## Abstract

**Introduction:**

At the request of the state, our burn center updated and rewrote the State Mass Burn Casualty Plan (MBCP) with a specific emphasis on helping local hospitals prepare to care for burn patients longer than the normal 96 hours. Grants funded outreach events to educate the various entities in the health regions on the new MBCP and practice implementing it in tabletop exercises (TTE). The goal of the TTE is to test the new state MBCP and identify barriers to successful implementation of the plan in a mock region-specific Burn Mass Casualty Disaster (BMCD).

**Methods:**

Two outreach events for three regions were performed. On 4/7/23 at 0820, an event for region 6 & 9 occurred on Teams with 20 participants. The second event, for region 2, took place on 4/14/2023 at 0800 at a county fairground with 16 participants.

Each event began by explaining the new MBCP, focusing on education, triaging, and caring for burn patients for >96 hrs. until transfers became available. Next was the TTE in which participants responded to a mock region-specific BMCD. The phases of the TTE included: Phase 1 (first 30 minutes), Phase 2 (30 minutes-2 hours), Phase 3 (up to 96 hours) and Phase 4 (96+hrs). The participants decided who and how the different entities would respond during each phase. All actions and communication were informed by real-time data acquired from the hospitals’ nursing supervisors and the state’s database capacity system.

**Results:**

The results are broken down by region. Region 2 is urban. The TTE lasted 4 hours. The scenario involved an explosion at a chemical plant during the day shift. The chemical plant and local EMS are immediately overwhelmed and call for help. 7 hospitals, on average 29 miles from the accident can offer support.

Region 6&9 are rural or frontier. The TTE lasted 193 minutes. A small plane crashes into a parade. Multiple burns and traumas are caused from the crash, leaking fuel, and burning parade wagons. Local EMS are immediately overwhelmed. 9 hospitals, on average 102 miles from the accident can offer support.

All regions experienced similar problems: difficulty establishing control at the scene and the initial responding hospital, insufficient decontamination supplies, traffic and delays in transporting patients necessitating the holding of burn patients longer than 96 hrs., and lack of staffing.

**Conclusions:**

Since both regions, despite their differences, share the same issues, this suggests that hospitals must update their internal and external disaster plans to address the problems listed above.

**Applicability of Research to Practice:**

When updating BMCP, consider adding a section where all regions incorporate mitigation plans for the expected issues: establishing scene control, decontamination, transportation of patients and delays, and staffing.

